# Effects of Soybean Oil Body as a Milk Fat Substitute on Ice Cream: Physicochemical, Sensory and Digestive Properties

**DOI:** 10.3390/foods11101504

**Published:** 2022-05-22

**Authors:** Wan Wang, Min Wang, Cong Xu, Zhijing Liu, Liya Gu, Jiage Ma, Lianzhou Jiang, Zhanmei Jiang, Juncai Hou

**Affiliations:** College of Food Science, Northeast Agricultural University, Harbin 150030, China; 13159806631@163.com (W.W.); 18246037810@163.com (M.W.); 15636116265@163.com (C.X.); liuzhijing22@126.com (Z.L.); andrea_hh@163.com (L.G.); jiage_ma@neau.edu.cn (J.M.); jlzname@neau.edu.cn (L.J.); zhanmeijiang@neau.edu.cn (Z.J.)

**Keywords:** soybean oil body, ice cream, melting properties, texture, sensory evaluation, digestive properties

## Abstract

Soybean oil body (SOB) has potential as a milk fat substitute due to its ideal emulsification, stability and potential biological activity. In this study, SOB was used as a milk fat substitute to prepare ice cream, expecting to reduce the content of saturated fatty acid and improve the quality defects of ice cream products caused by the poor stability of milk fat at low temperatures. This study investigated the effect of SOB as a milk fat substitute (the substitution amount was 10–50%) on ice cream through apparent viscosity, particle size, overrun, melting, texture, sensory and digestive properties. The results show SOB substitution for milk fat significantly increased the apparent viscosity and droplet uniformity and decreased the particle size of the ice cream mixes, indicating that there were lots of intermolecular interactions to improve ice cream stability. In addition, ice cream with 30% to 50% SOB substitution had better melting properties and texture characteristics. The ice cream with 40% SOB substitution had the highest overall acceptability. Furthermore, SOB substitution for milk fat increased unsaturated fatty acid content in ice cream and fatty acid release during digestion, which had potential health benefits for consumers. Therefore, SOB as a milk fat substitute may be an effective way to improve the nutritional value and quality characteristics of dairy products.

## 1. Introduction

Ice cream has good flavor and texture as a consumer-friendly frozen dairy product. Ice cream, as an oil-in-water frozen aerated emulsion, contains partially agglomerated fat globules, unfrozen viscous whey, ice crystals and air bubbles [1]. Generally, ice cream contains 10–16% fat, which is an important ingredient in ice cream and affects the melting resistance, shape retention and smoothness of ice cream after the freezing process [2]. Milk is usually the main raw material for preparing ice cream, containing about 3.5–5.0% fat, which mainly contains 98% triacylglycerol and a small amount of phospholipids, free fatty acids and cholesterol [3,4]. Interestingly, milk fat is triacylglycerol present in milk as an emulsion (oil-in-water), commonly known as fat globules. The particle sizes of fat globules range from 0.1 to 15 µm. Due to the wider particle size distribution, milk fat stored at low temperatures could cause aggregation of fat globules as fat crystals protrude from the globule surface and damage the fat globule membrane, resulting in poor performance defects in whole milk ice cream stability [5]. In addition, milk fat is associated with many negative health effects, mainly due to the relatively high content of saturated fatty acids, which could lead to the increase of cholesterol and low-density lipoprotein, thereby increasing the risk of cardiovascular disease [3]. Furthermore, the increasing consumer preference for natural and healthy functional foods has prompted ice cream makers to look for innovations in health-beneficial ingredients to meet consumer demand [6,7,8].

Soybean oil body (SOB) is lipid-storing organelles in soybean with a particle size in the approximate range of 0.4–2.0 μm [9]. The purified SOB consists mainly of neutral lipid droplets (87–91.89%) surrounded by natural emulsifiers, mainly phospholipids and basic proteins. SOB is endowed with unique stability and emulsifying properties owing to the charge of the surface proteins of SOB and the polarity of the phospholipids that increase the mutual repulsion between the SOBs to prevent aggregation [10]. In addition, SOB is rich in nutrients such as vitamin E, unsaturated fatty acids and phytosterols [10,11]. Among them, palmitic acid, oleic acid, α-linolenic acid and linoleic acid are the main unsaturated fatty acids in SOB, amounting to around 96% of the total fatty acids [12]. Typically, SOB is extracted and concentrated into a white cream that can be diluted to form a natural oil-in-water emulsion, and SOB retains its natural function and physical stability under processing conditions. SOB is rich in a variety of bioactive components, and ideal emulsification and stability, therefore it has the potential to be used as a milk fat substitute in various foods (e.g., ice cream, mayonnaise) [13].

In this study, SOB was served as a milk fat substitute to prepare ice cream, expecting to reduce the content of saturated fatty acid and improve the quality defects of ice cream products caused by the poor stability of milk fat at low temperatures. The apparent viscosity, particle size, melting properties, chemical composition, microstructure and digestive properties of ice cream were characterized to explore the consequence of SOB as a milk fat substitute on ice cream quality. While improving the beneficial ingredients and quality of ice cream to further determine the application value of SOB in ice cream, this study provides a theoretical underpinning for expanding the application of SOB in the food industry and also supplies innovative ideas for the development of healthy frozen foods.

## 2. Materials and Methods

### 2.1. Materials

Soybeans were obtained from the Soybean Institute of Northeast Agricultural University (Harbin, China). Skimmed milk powder was purchased from Inner Mongolia Yili Industrial Group Co., Ltd. (Hohhot, China). Vast cream was purchased from Qingdao Nestle Co., Ltd. (Qingdao, China).

### 2.2. Preparation of SOB

The preparation method of SOB referred to the description of Zhou et al., [9]. Briefly, after soaking clean soybeans in deionized water for 12 h, a 20% sucrose solution was added. The mixture was ground (18,000 r/min 120 s) and filtered to obtain soybean milk. Then, the soybean milk was centrifuged (8000× *g*, 4 °C, 20 min) to collect the precipitation. The precipitation was resuspended in 20% sucrose solution and centrifuged (8000× *g*, 4 °C, 20 min). This step was repeated three times to obtain SOB and sterilized at 100 °C for 20 min.

### 2.3. Preparation of Ice Cream

Skimmed milk powder (14%, *w*/*w*), granulated sugar (10%, *w*/*w*), egg yolk (5%, *w*/*w*), cream (12%, *w*/*w*) and water were mixed and filtered. Among them, SOB was used to replace cream according to 0% (control group), 10%, 20%, 30%, 40% and 50% of the amount of cream. Then, the above mixture was homogenized (15 MPa, 65 °C), pasteurized (75 °C, 20 min) and aged (4 °C, 12 h) to obtain the ice cream mixes. Finally, the ice cream mixes were whipped and frozen for 30 min and hardened (−18 °C, 24 h) to obtain ice cream samples.

### 2.4. Steady Shear Rheological Properties of Ice Cream Mixes

The method for the determination of the steady shear rheological properties of ice cream mixes referred to the description of Jiang et al., and was modified [14]. The steady shear rheological properties of the ice cream mixes were determined at 4 °C by the rotational rheometer (MARS40, Thermo, Waltham, MA, USA). The ice cream mixes were placed on the plate system (60 mm in diameter) of the rheometer. The strain was set to 0.6%. The range of the shear rate was 1–100 s^−1^ in 120 s to measure the apparent viscosity, consistency index (K) and flow behavior index (n).

### 2.5. Particle Size of Ice Cream Mixes

The measuring method of average particle diameter referred to the report of Zhao et al., [15]. The ice cream mixes were diluted 100-fold with SDS solution (1%, *w*/*v*) and measured by laser particle size distribution analyzer (HYL-1076, Haoyu Technology, Dandong, China). Droplet size measurements were reported as surface average diameter (D_[3,2]_), volume average diameter (D_[4,3]_) and median diameter (D_50_).

### 2.6. Overrun of Ice Cream

The overrun of ice cream was measured according to the method of Pon et al., [16]. Equal volumes of ice cream mixes and ice cream samples were weighed to calculate ice cream overrun. The formula for calculating the overrun was as follows, where the mass of the ice cream mixes was recorded as W_0_ and the mass of the ice cream sample was recorded as W_1_:

Overrun (%) = 100 × (W_0_ − W_1_)/W_1_ (1)

### 2.7. Melting Properties of Ice Cream

The determination method of the melting properties referred to the description of Kurt et al., [1]. The ice cream samples were placed on a metal mesh screen at 37 °C for 60 min to determine the first dripping time (min) and the ice cream melting rate (%).

### 2.8. Texture of Ice Cream

The hardness, adhesiveness, springiness and chewiness of ice cream samples were determined by texture analyzer (TA-XT Plus, SMATA, Godalming, England). The specific parameters are as follows: the texture analyzer probe was P/5, the probe diameter was 5 mm, the pre-measurement rate was 8 mm/s, the mid- and post-measurement rate was 2 mm/s, the penetration depth was 15 mm and the trigger force was 20 g.

### 2.9. Physicochemical Properties of Ice Cream

The chemical composition of the ice cream was determined according to AOAC [17]. The content of fatty acids was determined by gas chromatography–mass spectrometry (Nexis GC-2030, Shimadzu, Kyoto, Japan) [18]. The chromaticity of ice cream samples was measured by color difference meter (ZE6000, Nippon Denshoku, Tokyo, Japan), in which L* value represented the light dark value, a* represented the red green value and b* represented the yellow blue value. The flavor of the ice cream samples was determined by electronic nose.

### 2.10. Microstructure of Ice Cream

The observation method of ice cream microstructure referred to the report of Zhou et al., and was modified [19]. The microstructures of ice cream were observed using ultra-high distraction microscopy (Deltavision OMX SR, GE, Boston, MA, USA). An amount of 2 mL of diluted 5 times ice cream samples were added with 40 μL of Nile red (0.1%) and 50 μL Nile blue (0.1%) (Amresco, Washington, DC, USA) and then avoided light reaction for 30 min. An amount of 1.5 μL of the reaction liquid was placed in a slide and then the microstructure and the distribution of fat and protein were observed at the excitation wavelength of 488 nm.

### 2.11. Sensory Evaluation of Ice Cream

The sensory evaluation system is established by using the fuzzy mathematical model. The sensory scoring standard was shown in Table S1 in the Supplementary Materials.

Then, according to the sensory evaluation index, the index set was established. U = {u_1_, u_2_, u_3_, u_4_}, where u_1_, u_2_, u_3_ and u_4_ represented the color, taste, texture and flavor of ice cream, respectively. The sensory evaluation comment set was established V = {v_1_, v_2_, v_3_, v_4_}, where v_1_, v_2_, v_3_ and v_4_ represented excellent, good, medium and poor, respectively. The middle score of each grade was selected as the final sensory score, V = {9, 7, 5, 3}.

In addition, 10 professionally trained sensory panelists (half male and half female) performed a weighted analysis on the color, taste, texture and flavor of ice cream samples. The frequency statistics method was adopted to determine the weight of each evaluation index. The weight set was X = {x_1_, x_2_, x_3_, x_4_}.

Furthermore, the fuzzy matrix was established. The 10 sensory panelists scored ice cream samples based on color, taste, texture and flavor. The final votes for each grade (excellent, good, medium, poor) were counted. Fuzzy matrix R was the number of votes at each grade divided by the total number of sensory panelists. Fuzzy relation evaluation set was Y = X × R. Sensory score was T = Y × V.

### 2.12. Simulated Digestion of Ice Cream In Vitro

Preparation of artificial saliva: 15.1 mmol KCl, 3.7 mmol KH_2_PO_4_, 13.6 mmol NaHCO_3_, 0.15 mmol MgCl_2_(H_2_O)_6_, 0.06 mmol (NH_4_)_2_CO_3_, 0.075 mmol CaCl_2_(H_2_O)_2_ and 50 mg ptyalin (Shanghai Yuanye Biotechnology Co., Ltd., Shanghai, China) were dissolved in deionized water with constant volume to 500 mL and adjusted pH to 6.8.

Preparation of artificial gastric juice: 6.9 mmol KCl, 0.9 mmol KH_2_PO_4_, 25 mmol NaHCO_3_, 47.2 mmol NaCl, 0.1 mmol MgCl_2_(H_2_O)_6_, 0.5 mmol (NH_4_)_2_CO_3_, 0.075 mmol CaCl_2_(H_2_O)_2_ and 10^6^ U pepsin were dissolved in deionized water with constant volume to 500 mL and adjusted pH to 2.0.

Preparation of artificial intestinal juice: 6.8 mmol KCl, 0.8 mmol KH_2_PO_4_, 85 mmol NaHCO_3_, 38.4 mmol NaCl, 0.33 mmol MgCl_2_(H_2_O)_6_, 0.3 mmol CaCl_2_(H_2_O)_2_ 160 mmol bile salt and 5 *×* 10^4^ U trypsin were dissolved in deionized water with constant volume to 500 mL and adjusted pH to 7.0.

The digestion model in vitro was constructed according to the method of Ma et al., [20]. Briefly, ice cream samples were mixed with artificial saliva (1:1, *v*/*v*) and digested for 5 min at 100 rpm/min on a 37 °C incubator shaker. Then, the artificial saliva digesta was mixed with artificial gastric juice (1:1, *v*/*v*) and digested for 60 min at 100 rpm/min on a 37 °C incubator shaker. Finally, the artificial gastric digesta was mixed with artificial intestinal juice (1:1, *v*/*v*) and digested for 120 min at 100 rpm/min on a 37 °C incubator shaker. Finally, the digesta were placed at −80 °C to terminate the reaction and stored. In addition, the artificial saliva, artificial gastric juice and artificial intestinal juice were replaced with equal volumes of deionized water in the control sample (undigested ice cream dilution sample).

### 2.13. Determination of Free Fatty Acid

The pH-stat method was used to measure the content of the free fatty acids released in simulated intestinal juice digestion (20, 40, 60, 80, 120 min) according to the method of Hageman et al., [21]. Changes in the pH of the digesta were due to the release of free fatty acids during digestion, so the content of free fatty acids can be determined by recording the consumption of NaOH to neutralize the pH of the digesta. The formula for calculating the concentration of free fatty acid was as follows, where the volume of NaOH consumed when the pH of the digesta reached 7.0 was recorded as V_1_ (μL), the concentration of NaOH was recorded as C_1_ (mol/L) and the digest volume was recorded as V_2_ (mL):

Free fatty acid (μmol/mL) = (V_1_ × C_1_)/V_2_ (2)

### 2.14. Determination of Protein Digestibility

The determination method of protein digestibility followed the previous report of Li et al., [22]. An amount of 1 mL of trichloroacetic acid (10%) was added to 1 mL of gastric digesta, intestinal digesta and the control sample (undigested ice cream dilution sample), respectively. After mixing, the mixture was centrifuged at 20 °C for 15 min with a centrifugal force of 8000× *g* and the supernatant was taken. The protein content of each supernatant was then determined using the BCA protein assay kit. The protein content remaining in the digesta was recorded as C_1_ and the protein content in the control sample (undigested ice cream dilution sample) was recorded as C_2_. The formula for calculating the protein digestibility was as follows:

Protein digestibility (%) = 100 × (1 − C_1_/C_2_) (3)

### 2.15. Sodium Dodecyl Sulphate–Polyacrylamide Gel Electrophoresis

The experiment method referred to the description of Nikiforidis et al., and was improved [23]. Briefly, ice cream samples were treated with Tris-HCL buffer solution (6.25 mmol/L) and heated by water bath (100 °C, 10 min). After the electrophoretic, the gel was stained by Komas Blue R-250 and was discolored by glacial acetic acid

### 2.16. Statistical Analysis

All results were the mean of three independent replicates and expressed as mean ± standard deviation. One-way analysis of variance (ANOVA) was used with SPSS statistics software (SPSS 26.0, Chicago, IL, USA). *p <* 0.05 represented significant difference with Duncan’s test.

## 3. Results

### 3.1. Apparent Viscosity of Ice Cream Mixes

**Figure 1:**
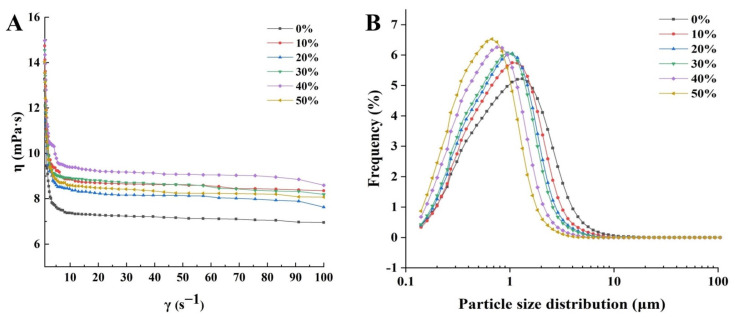
Effects of different SOB substitution amounts on (**A**) apparent viscosity and (**B**) particle size distribution of ice cream mixes.

The apparent viscosity is a crucial indicator reflecting the flow behavior and the taste of ice cream. The sample exhibited shear thinning behavior shown in Figure 1A, as the apparent viscosity of the ice cream mixes decreased with the increase of shear rate [1]. This was because the shearing of the ice cream mixes would produce a destructive effect, which increased the arrangement of constituent molecules in its structure, resulting in the non-Newtonian pseudoplastic flow of the ice cream mixes [24]. In addition, the consistency index (K) and flow behavior index (n) of the ice cream mixes were shown in Table 1. The n values of ice cream mixes were less than 1 and ranged from 0.54 to 0.65, indicating that the ice cream mixes were pseudoplastic fluids. Meanwhile, the K value of ice cream mixes increased significantly with the increase of SOB substitution amount, while the n value decreased significantly. When the SOB substitution amount was 40%, the K value reached the maximum and showed the lowest n value. The smaller value of n reflected a departure from the Newtonian flow, indicating higher pseudoplasticity of the ice cream mixes with 40% SOB substitution [25]. Moreover, the apparent viscosity of ice cream mixes increased significantly with the increase of SOB substitution amount. Possibly due to the excellent emulsion stability of SOB, high concentration of SOB could act as a stabilizer to maintain the emulsion system of ice cream, thereby increasing the apparent viscosity of SOB ice cream [26]. Furthermore, the increase of apparent viscosity and consistency index of the ice cream mixes with SOB as a milk fat substitute might be due to the differences in intermolecular interactions. Variations in interactions were caused by differences in biopolymer types and differences in the chemical composition of the surfaces of SOB and milk fat globules. As the substitution amount of SOB increased, the changes in aggregation degree of droplets and the chemical composition of fat globules in ice cream mixes led to altered intermolecular interactions. Biopolymers generate new ordered and improved structures through hydrogen bonding or hydrophobic interactions between non-polar side segments of the carbon backbone to cause differences in rheological behavior [27,28].

### 3.2. Particle Size of Ice Cream Mixes

The volume-weighted average particle size (D_[4,3]_) and particle size distribution of droplets are effective indicators for evaluating the stability of ice cream mixes and the degree of droplet coalescence and are important for improving the sensory properties of food products [29]. The particle size of the ice cream mixes showed a unimodal distribution and the particle size peak distribution of the ice cream mixes without SOB substitution was at 1.32 μm (Figure 1B). With the increase of SOB substitution amount, the particle size of ice cream slurry gradually decreased, especially when SOB replacement amount was 50%, the particle size peak distribution of ice cream slurry was at 0.67 μm. The SOB ice cream mixes had a smaller particle size compared with full milk fat ice cream mixes, probably because the particle size of SOB was less than the particle size of the milk fat globules. Therefore, as the SOB substitution increased, the particle size of the ice cream mixes gradually decreased [9]. On the other hand, as a natural emulsifier, SOB had an excellent stabilizing effect on the dispersion of droplets in ice cream mixes [30]. In addition, due to the surface protein and polarity phospholipids, the SOB surface could form a tight and stable charged layer, thereby increasing the spatial resistance between SOBs to avoid aggregation [10]. Therefore, the ice cream mixes with SOB substitution had a smaller particle size and particle dispersion index than the ice cream mixes without SOB substitution, indicating that the ice cream mixes with SOB substitution had a more stable emulsion system and showed a trend of increasing dependence on the SOB substitution amount [31]. In addition, the smaller particle size could enhance the creamy flavor characteristics of ice cream.

### 3.3. Overrun of Ice Cream

**Figure 2:**
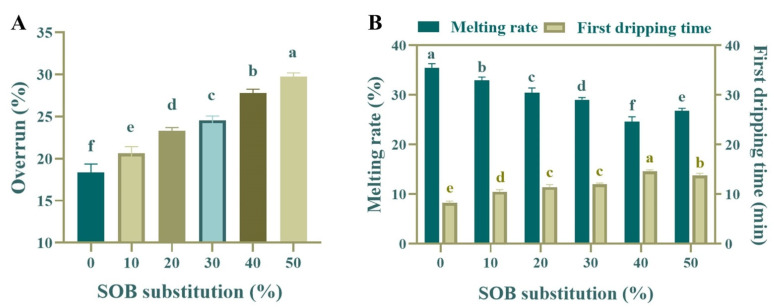
Effects of different SOB substitution amounts on (**A**) overrun and (**B**) melting properties of ice cream. Different alphabet represents significant difference in the same indicator (*p* < 0.05).

The overrun represents the air content in ice cream and is a crucial indicator affecting the melting, texture, and sensory characteristics of ice cream [1]. According to Figure 2A, the overrun of ice cream without SOB substitution was (18.37 ± 0.99)%, while when the SOB substitution amount was 40%, the overrun of ice cream reached the maximum, which was (29.74 ± 0.47)%. The overrun of ice cream with SOB substitution increased significantly (*p* < 0.05), which may be correlated with significantly higher foamability of SOB than milk fat globules. In addition, it may also be attributed to the increase in the apparent viscosity of the ice cream mix with increasing SOB substitution. According to reports, the apparent viscosity of the emulsion system had a crucial impact on the overrun of ice cream, because a certain apparent viscosity was required to produce moderate overrun [32]. The spherical bubbles in ice cream were usually surrounded by a network of partially coalesced fat droplets, which were also surrounded by whey protein, casein and emulsifiers; therefore, as a milk fat substitute, the addition of SOB increased the apparent viscosity of the ice cream mixes, which stabilized the formation of air bubbles. Meanwhile, the higher apparent viscosity allowed the air to form many smaller air chambers in the ice cream, giving the ice cream a higher overrun [33]. Furthermore, the increase in the apparent viscosity of the ice cream mixture caused by the increase in SOB substitution was conducive to preventing the collapse and coagulation of air bubbles in the ice cream [34].

### 3.4. Melting Properties of Ice Cream

The effects of different SOB substitution amounts on the melting rate and the first dripping time of ice cream were shown in Figure 2B. The melting rate of ice cream without SOB substitution was (35.50 ± 2.12)%, while when the SOB substitution amount was 40%, the melting rate of ice cream decreased to (24.57 ± 1.24)% (*p* < 0.05). Meanwhile, compared with the ice cream without SOB substitution for milk fat, the first dripping time of the ice cream with SOB substitution was significantly extended (*p* < 0.05). This may be because the overrun of the ice cream increased with the amount of SOB substitution, meaning there were more air chambers in the ice cream, which reduced the melt rate. Air is a bad conductor of heat, which will reduce the heat diffusion rate of ice cream, so the melting rate of ice cream will be decreased [35]. In addition, the increase in SOB substitution increased the apparent viscosity of the ice cream mixes, which could prevent the migration of water molecules in the ice cream at ambient temperature to decrease the fluidity of ice cream, thereby increasing the anti-meltdown of ice cream [27]. Furthermore, it has been reported that the degree of aggregation of the droplets in the ice cream was also significantly correlated with the melting rate of ice cream [36]. As the amount of SOB substituted for milk fat increased, the particle size and the particle distribution index of the ice cream mixes decreased, which could improve the stability of the ice cream emulsion system, thereby preventing the migration of water molecules and decreasing the melting rate of SOB ice cream [36]. In contrast, for the full milk fat ice cream with lower overrun and stability there was not enough network space structure to prevent melting and complete collapse. Therefore, the substitution of milk fat with SOB could improve the melting properties of full milk fat ice cream and the decrease of the melting rate was dependent on the increase of the substitution amount of SOB.

### 3.5. Texture Properties of Ice Cream

The effects of different SOB substitution amounts on the texture properties of ice cream were shown in Table 2. Compared with ice cream without SOB substitution, the hardness of SOB ice cream decreased significantly with the increase of SOB substitution amount, but the adhesiveness, springiness and chewiness increased significantly with the increase of SOB substitution amount (*p* < 0.05).

The hardness distinction between ice cream with SOB substituted and full milk fat ice cream may be that SOB substitution increased the apparent viscosity of the ice cream mixes. The increased viscosity of the ice cream network structure could decrease the formation of bulky and large-sized ice crystals during freezing, which would decrease the hardness of the ice cream [37]. This result was similar to the report of Kurt et al., that the addition of quince seed increased the viscosity of ice cream mixes, which led to a decrease in hardness of ice cream [1]. In addition, the ice cream with 50% SOB substitution had high adhesiveness, springiness and chewiness, which indicated that the addition of SOB improved the recovery rate of ice cream after deformation and signified that the yield stress of ice cream increased [1]. In general, there is a significant correlation between oral processing parameters and textural properties (i.e., adhesiveness, springiness and chewiness) [38]. The ice cream with SOB substitution will have a smoother mouthfeel during tasting due to the increase in ice cream adhesiveness and the decrease in ice crystal size [39]. In addition, the food with high springiness has reversible deformation during chewing, and chewiness is generally positively correlated with the number of chews before swallowing [38]. Therefore, the chewiness and springiness of ice cream increased with SOB substitution, which could prolong the residence time of ice cream in the mouth, which was important for increasing the oral perception of milk and soy flavors.

### 3.6. Physicochemical Analysis of Ice Cream

The effects of different SOB substitution amounts on the physicochemical analysis of ice cream were shown in Table 3. The contents of total soluble solids (TSS), protein and carbohydrate in ice cream with SOB substitution increased significantly and the content of fat decreased significantly (*p* < 0.05) compared with ice cream without SOB substitution, indicating that there were differences in the chemical composition of SOB and milk fat. This is because milk fat globules contain 98% triacylglycerols and less than 2% protein, while SOB contains 87–91.89% neutral lipid droplets and 5.42–13% basic protein [3,10]. In addition, the content of saturated fatty acids in ice cream with SOB substitution for milk fat decreased significantly and the content of unsaturated fatty acids increased significantly compared with full milk fat ice cream (*p* < 0.05), therefore the contents of saturated fatty acids and unsaturated fatty acids in SOB and milk fat globule was different. Milk fat mainly contained saturated fatty acids, while the SOB mainly contained unsaturated fatty acids [40]. More evidence shows that a large amount of myristic acid intake would increase the risk of plasma cholesterol and cardiovascular disease [41]. In contrast, a diet rich in unsaturated fatty acids has proven to improve high density lipoprotein function that protects patients with cardiovascular disease [42]. Meanwhile, unsaturated fatty acids play a crucial part in improving immune function and reducing systemic inflammation by regulating patterns on immune cells [43]. Therefore, the ice cream prepared with SOB substitution for milk fat had potential and healthy functions.

### 3.7. Color Properties of Ice Cream

The effects of different SOB substitution amounts on the color properties of ice cream were shown in Table 3. The a* value in ice cream with SOB substitution significantly decreased with the increase of SOB substitution amount compared with ice cream without SOB substitution (*p* < 0.05), which was due to the difference in the color properties of SOB and milk fat itself, indicating that SOB has less red than the milk fat. Additionally, it could be due to the decreased particle size of the droplets, which scattered the light, making the ice cream whiter [44]. However, the difference between the L* and b* values of ice cream with different SOB substitution amounts was not significant (*p* > 0.05). In addition, the ΔE value of the ice cream with different SOB substitution amounts was 1.60, 0.70, 0.99, 1.97 and 2.91. Bayram et al., [45] reported that when the ΔE value reached 3.7, the naked eye could perceive the difference in color. Therefore, the ice cream with SOB substitution for milk fat cannot bring unpleasant color properties to the naked eye.

### 3.8. Microstructure of Ice Cream

**Figure 3:**
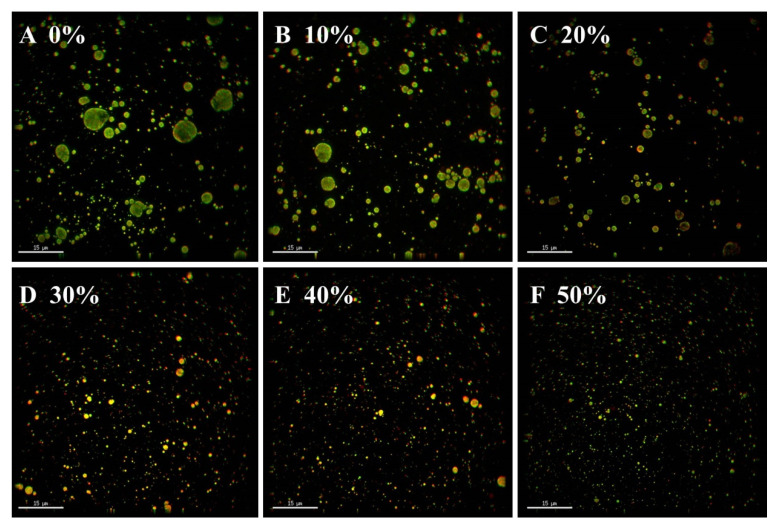
Effects of different SOB substitution amounts on microstructure of ice cream. (**A**–**F**) represent the ice cream with 0%, 10%, 20%, 30%,40% and 50% of SOB substitution for milk fat, respectively.

Ultra-high-resolution optical microscope was used to observe the microstructure of ice cream as shown in Figure 3 (green represented fat and red represented protein). The droplets of ice cream without SOB substitution for milk fat aggregated and formed larger particles. However, the droplets of ice cream with SOB substitution were dispersed and uniform, and the particle size of the droplets in the ice cream gradually decreased with the increase of the SOB substitution amounts. This may be due to the small particle size and the high stability of SOB, so there are few droplet aggregates formed [36]. In addition, due to the surface protein and polarity phospholipids, the SOB surface could form a tight and stable charged layer, thereby increasing the spatial resistance between SOBs to avoid aggregation [10]. The above results indicated that SOB has an excellent stabilizing effect on the dispersion of droplets in ice cream. This result was consistent with the decrease in particle size and particle distribution index of the ice cream mixes with the increase in SOB substitution.

### 3.9. Flavor Properties of Ice Cream

**Figure 4:**
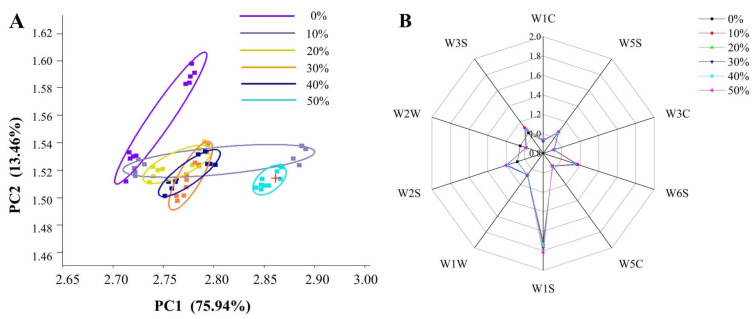
Effects of different SOB substitution amounts on flavor properties of ice cream, they should be listed as: (**A**) principal component analysis plot; (**B**) radar chart.

The electronic nose signals of ice cream with different SOB substitution amounts were analyzed to distinguish the flavor differences. The principal component analysis (PCA) chart (Figure 4A) showed that the contribution rate of the first principal component of the ice cream sample was 75.94%, the contribution rate of the second principal component was 13.46% and the cumulative contribution rate was 89.40%, indicating that the sample information was sufficient [46]. In addition, the radar chart (Figure 4B) showed W1S (methyls), W2S (alcohols, aldehydes and ketones), W3S (long-chain alkanes) and W6S (hydrides) in the volatile flavor compounds of ice cream majored contribution. Among them, the response intensity of W2S in ice cream with SOB substitution for milk fat was higher than that in ice cream without SOB substitution and the response intensity of W2W (sulfides) was lower than that in ice cream without SOB substitution. This may be due to the difference in flavor compounds in SOB and milk fat, illustrating that ice cream with SOB substitution had higher abundances of alcohols and aromatics compounds and lower abundances of sulfides compounds than full milk fat ice cream. However, except for the ice cream with 50% SOB substitution, the PCA chart of the ice cream samples with a different SOB substitution amount were overlapped, which was due to the smaller difference between the response values of the electronic nose sensor for different ice cream samples. The reason for the difference in flavor properties between the ice cream with 50% SOB substitution and the other ice cream samples may be that the soy flavor of the 50% SOB significantly masked the creamy flavor. Furthermore, except for W2W and W2S, the response values of other sensors were similar between the full milk fat ice creams and the ice cream with SOB substitution. The results indicated that the SOB substitution of less than 50% could maintain the original flavor of full milk fat ice cream while imparting proper bean flavor.

### 3.10. Sensory Score of Ice Cream

Table 4 showed the scoring of ice cream with different SOB substitution amounts for 10 sensory panelists. Then, based on the fuzzy mathematical sensory evaluation model, the comprehensive score matrix was established to calculate the sensory score. According to the sensory panelists, the ice cream with SOB substituted for milk fat had a distinct soybean flavor and this flavor increased with SOB substituted amounts without negatively affecting sensory scores. The results showed that 40% SOB substitution improved the sensory score (8.46) of ice cream, indicating improved acceptability. In addition, although the sensory scores of ice cream with 50% SOB substitution and with 40% SOB substitution were not significantly different, but, according to the sensory evaluation panelists, the bean flavor of ice cream with 50% SOB substitution obviously masked the creamy flavor, so the creamy flavor was not strong enough to make ice cream with 50% SOB substitution less acceptable than ice cream with 40% SOB substitution. Moreover, according to the sensory evaluation panelists, ice cream substituted with 30–50% SOB had a smooth texture and uniform melting compared with full milk fat ice cream, which was consistent with the texture property results in this study. This may be due to the particle size and particle distribution index of ice cream gradually decreasing with the increase of SOB substitution, indicating that the droplets in the ice cream were uniformly dispersed and easily melted uniformly in the mouth. Furthermore, the smooth mouthfeel indicated that SOB substitution for milk fat eliminated the roughness of the full milk fat ice cream, suggesting that the increase in SOB substitution decreased the ice crystals detectable in the ice cream during melting [37]. This result was consistent with the change trends of ice cream hardness. The sensory evaluation results showed that the ice cream with 40% SOB substitution for milk fat had the highest sensory score, even more generally acceptable than the full milk fat ice cream.

### 3.11. Particle Size of Ice Cream In Vitro Digestion

**Figure 5:**
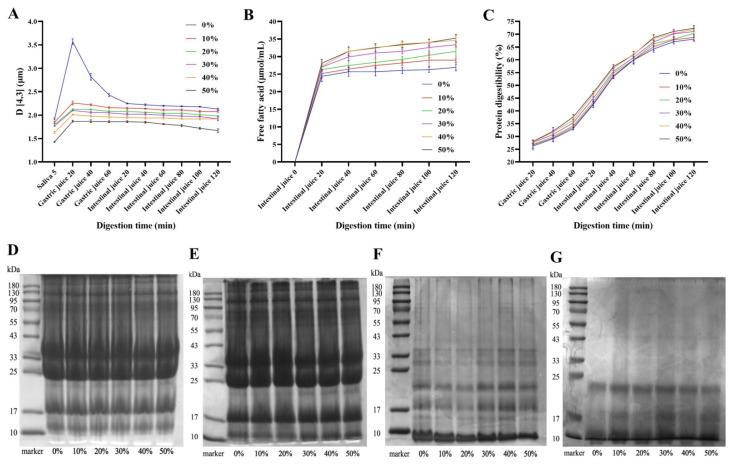
Effects of different SOB substitution amounts on digestive properties of ice cream, they should be listed as: (**A**) average particle size; (**B**) free fatty acid; (**C**) protein digestibility; (**D**) not digested; (**E**) digestive in artificial saliva for 5 min; (**F**) digestive in artificial gastric juice for 60 min; (**G**) digestive in artificial intestinal juice for 120 min.

The particle size of ice cream in vitro digestion was shown in Figure 5A. With the extension of digestion time, the particle size of ice cream digesta showed a trend of first increasing and then decreasing. During simulated saliva digestion, the D_[4,3]_ of ice cream increased significantly, which may be due to the binding of anionic mucins in saliva to the positive charges of droplet surface proteins, depleting the interaction between droplets and causing aggregation, resulting in the increase of D_[4,3]_ [47]. In addition, the D_[4,3]_ of ice cream digesta increased significantly when digested in simulated gastric juice for 20 min. This result was because the gastric juice was acidic and contained various protein hydrolase, thus the casein micelles and fat globule surface proteins in ice cream samples were denaturation, hydrolyzed and aggregated [47]. Meanwhile, the interface protein of the droplet was partially hydrated, resulting in a decrease in electrostatic exclusion, i.e., flocculation, and the D_[4,3]_ of the digesta increased [48]. In particular, in simulated gastric juice for 20 min, the D_[4,3]_ of the full milk fat ice cream digesta was significantly higher than that of the ice cream with SOB substitution, which may be because the surface protein of milk fat globules was more easily hydrolyzed by pepsin than the surface protein of SOB, thereby destabilizing the ice cream emulsion system, resulting in digesta particle size increase. Furthermore, with the increase of digestion time in gastric and intestinal juice, the flocculated protein was decomposed by protease, resulting in the decrease of D_[4,3]_ of ice cream digesta [47]. Among them, the D_[4,3]_ of ice cream digesta with SOB substitution was always smaller than that of full milk fat ice cream digesta and the D_[4,3]_ of digesta decreased with the increase of SOB substitution amount. This meant that the ice cream with SOB substitution was easier to digest and absorb than full milk fat ice cream.

### 3.12. Free Fatty Acids of Ice Cream In Vitro Digestion

The intestine is the main place for fat digestion and release, which transforms fat into absorption forms through interaction with pancreatic and bile secretions [3]. The free fatty acid release of ice cream in vitro digestion was shown in Figure 5B. With the movement of digestion time, the free fatty acid releases showed the trend of increased first and then gentle. This is because the triacylglycerols were converted into free fatty acids under the action of trypsin and bile salts when the ice cream gastric digesta was digested in simulated intestinal juice for 20 min, thus significantly increasing the release of free fatty acids. Furthermore, at the same digestion time, the release of free fatty acids from the ice cream digesta increased significantly with increasing SOB substitution. This may be due to the smaller particle size of droplets of ice cream with SOB substitution, so the same volume of fat had a larger surface, which could promote lipase to enter the oil–water interface to convert triglycerides into free fatty acids and glycerol, resulting in free fatty acids release in digestive juices increased [49,50]. The results indicated that the ice cream prepared by substitution SOB for milk fat was easier to digest and release fatty acids than the whole milk fat ice cream, which was more conducive to the intake of nutrients by the human body.

### 3.13. Protein Digestibility of Ice Cream In Vitro Digestion

The protein digestibility of ice cream in vitro digestion was shown in Figure 5C. With the increase of digestion time, the digestibility of protein increased significantly and the protein digestibility of samples in intestinal juice was significantly higher than that in gastric juice. This may be due to the fact that trypsin in intestinal juice is more likely to break peptide bonds than pepsin in gastric juice [51]. This result was consistent with the decreased protein molecular weight after simulated gastric and intestinal digestion shown in Figure 5F,G. Furthermore, in addition, the protein digestibility of ice cream samples increased significantly with the SOB substitution and they were higher than that of full milk fat ice cream, which may be due to the difference in the composition of SOB and milk fat globule surface proteins, that is, SOB surface proteins are more easily hydrolyzed by protease than milk fat globule surface proteins. Moreover, it may also be because the ice cream with SOB substitution had a smaller particle size, so the droplet surface protein had a larger contact surface area with the protease, thus improving the protein digestibility [49]. Therefore, the results showed that the ice cream prepared with SOB as a milk fat substitute was easier to digest than the protein in full milk fat ice cream, which had a positive contribution to the digestion and absorption of ice cream by the human body.

## 4. Conclusions

This study provided new insights into ice cream prepared with SOB as a milk fat substitute. SOB substitution for milk fat not only decreased the content of saturated fatty acids in ice cream, but also improved the quality defects of ice cream products caused by the poor stability of milk fat at low temperatures. SOB substitution for 40–50% milk fat could improve the stability of ice cream by increasing the apparent viscosity and decreasing the particle size of the ice cream mixes, which made the ice cream form the desired quality. Therefore, ice cream prepared substitution 40–50% SOB for milk fat had ideal physicochemical properties and potential biological activity. Among them, the ice cream prepared substitution 40% SOB for milk fat had the best sensory acceptability and had the potential for practical application. This study provided a significant theoretical underpinning for the application of SOB in dairy products.

## Figures and Tables

**Table 1:** Effects of different SOB substitution amounts on consistency index, flow behavior index, particle size and uniformity of ice cream mixes.

		SOB Substitution Amounts (%)					
		0	10	20	30	40	50
Rheological	K (Pa·s^n^)	0.22 ± 0.01 ^e^	0.32 ± 0.02 ^b^	0.26 ± 0.01 ^d^	0.31 ± 0.01 ^b^	0.36 ± 0.01 ^a^	0.29 ± 0.01 ^c^
	n	0.65 ± 0.02 ^a^	0.56 ± 0.01 ^d^	0.61 ± 0.02 ^b^	0.58 ± 0.01 ^c^	0.54 ± 0.01 ^e^	0.59 ± 0.02 ^bc^
	R^2^	0.9372	0.9396	0.9452	0.9403	0.941	0.9308
Particle size	D_50_ (μm)	1.04 ± 0.02 ^a^	0.92 ± 0.02 ^b^	0.84 ± 0.01 ^c^	0.80 ± 0.01 ^d^	0.63 ± 0.01 ^e^	0.58 ± 0.01 ^f^
	D_[4,3]_ (μm)	1.36 ± 0.10 ^a^	1.15 ± 0.03 ^b^	1.02 ± 0.01 ^c^	0.97 ± 0.01 ^d^	0.77 ± 0.02 ^e^	0.69 ± 0.03 ^f^
	D_[3,2]_ (μm)	0.72 ± 0.01 ^a^	0.67 ± 0.03 ^b^	0.63 ± 0.01 ^c^	0.60 ± 0.01 ^d^	0.50 ± 0.01 ^e^	0.46 ± 0.01 ^f^
	PDI	0.84 ± 0.03 ^a^	0.68 ± 0.01 ^b^	0.64 ± 0.00 ^c^	0.61 ± 0.01 ^d^	0.58 ± 0.01 ^e^	0.53 ± 0.02 ^f^

K—consistency index; n—flow behavior index; R^2^—correlation coefficient; PDI—particle dispersion index. Results are mean ± SD of three determinations. Different alphabet represents significant difference in the row (*p* < 0.05).

**Table 2:** Effects of different SOB substitution amounts on the texture properties of ice cream.

	SOB Substitution Amounts (%)					
	0	10	20	30	40	50
Hardness (g)	5899.93 ± 56.69 ^a^	5506.79 ± 92.58 ^b^	5042.31 ± 130.08 ^c^	4740.50 ± 96.28 ^d^	4326.72 ± 79.66 ^e^	4223.43 ± 47.14 ^f^
Adhesiveness (g·s)	138.97 ± 2.80 ^f^	165.94 ± 4.59 ^e^	221.68 ± 1.78 ^d^	256.21 ± 6.72 ^c^	270.37 ± 9.10 ^b^	315.96 ± 4.35 ^a^
Springiness	0.73 ± 0.02 ^d^	0.78 ± 0.01 ^c^	0.80 ± 0.01 ^bc^	0.81 ± 0.01 ^b^	0.82 ± 0.01 ^ab^	0.84 ± 0.04 ^a^
Chewiness	243.18 ± 5.15 ^e^	263.56 ± 10.98 ^d^	314.82 ± 4.80 ^c^	346.46 ± 6.96 ^b^	384.99 ± 4.10 ^a^	392.91 ± 2.14 ^a^

Results are mean ± SD of three determinations. Different alphabet represents significant difference in the row (*p* < 0.05).

**Table 3:** Effects of different SOB substitution amounts on the physicochemical parameters of ice cream.

		SOB Substitution Amounts (%)					
		0	10	20	30	40	50
Physicochemical properties	TSS °Bx	30.65 ± 0.03 ^f^	30.81 ± 0.04 ^e^	30.87 ± 0.02 ^d^	30.97 ± 0.03 ^c^	31.04 ± 0.02 ^b^	31.08 ± 0.01 ^a^
	Fat %	5.97 ± 0.04 ^a^	5.88 ± 0.03 ^b^	5.78 ± 0.04 ^c^	5.71 ± 0.05 ^d^	5.63 ± 0.02 ^e^	5.53 ± 0.03 ^f^
	Protein %	5.57 ± 0.03 ^f^	5.68 ± 0.04 ^e^	5.76 ± 0.03 ^d^	5.84 ± 0.05 ^c^	5.92 ± 0.04 ^b^	6.01 ± 0.02 ^a^
	Carbohydrate %	18.14 ± 0.06 ^f^	18.25 ± 0.05 ^e^	18.34 ± 0.06 ^d^	18.42 ± 0.03 ^c^	18.48 ± 0.02 ^b^	18.53 ± 0.04 ^a^
Fatty acid content	Myristic acid %	5.92 ± 0.01 ^a^	5.34 ± 0.03 ^b^	4.96 ± 0.08 ^c^	4.51 ± 0.10 ^d^	4.31 ± 0.04 ^e^	3.79 ± 0.03 ^f^
	Hyperic acid %	27.25 ± 0.11 ^a^	26.40 ± 0.03 ^b^	25.61 ± 0.02 ^c^	24.25 ± 0.60 ^d^	23.93 ± 0.22 ^e^	22.54 ± 0.17 ^f^
	Glycolic acid %	9.08 ± 0.08 ^a^	8.64 ± 0.00 ^b^	8.36 ± 0.10 ^b^	8.06 ± 0.19 ^c^	7.95 ± 0.18 ^c^	7.40 ± 0.04 ^d^
	Oleic acid %	25.65 ± 0.06 ^a^	25.90 ± 0.01 ^a^	25.93 ± 0.32 ^a^	25.98 ± 0.51 ^a^	26.10 ± 0.33 ^a^	26.20 ± 0.08 ^a^
	Linoleic acid %	4.78 ± 0.03 ^f^	7.90 ± 0.04 ^e^	11.13 ± 0.12 ^d^	14.48 ± 0.20 ^c^	15.97 ± 0.01 ^b^	17.95 ± 0.13 ^a^
	Linolenic acid %	0.34 ± 0.01 ^f^	0.96 ± 0.03 ^e^	1.40 ± 0.04 ^d^	1.85 ± 0.01 ^c^	2.05 ± 0.02 ^b^	2.31 ± 0.01 ^a^
Color properties	L*	95.95 ± 2.10 ^a^	97.40 ± 0.59 ^a^	96.60 ± 0.56 ^a^	96.86 ± 2.02 ^a^	97.90 ± 1.31 ^a^	98.83 ± 3.87 ^a^
	a*	0.64 ± 0.02 ^a^	0.56 ± 0.02 ^b^	0.41 ± 0.05 ^c^	0.45 ± 0.03 ^c^	0.35 ± 0.03 ^d^	0.30 ± 0.02 ^e^
	b*	21.31 ± 0.27 ^ab^	20.65 ± 0.05 ^b^	21.42 ± 0.08 ^a^	20.97 ± 0.17 ^ab^	21.32 ± 0.33 ^ab^	21.52 ± 0.79 ^a^
	ΔE	-	1.60	0.70	0.99	1.97	2.91

TSS—total soluble solids; L*—lightness; a*—redness; b*—yellowness; ΔE—chromatic aberration. Results are mean ± SD of three determinations. Different alphabet represents significant difference in the row (*p* < 0.05).

**Table 4:** Effects of different SOB substitution amounts on the sensory score of ice cream.

SOB Substitution Amounts (%)	Color				Taste				Texture				Flavor				Sensory Score
	E	G	M	P	E	G	M	P	E	G	M	P	E	G	M	P	
0	7	3	0	0	5	3	2	0	6	3	1	0	7	3	0	0	8.06 ± 0.19 ^bc^
10	7	3	0	0	2	5	2	1	4	4	1	1	6	3	1	0	7.44 ± 0.19 ^d^
20	7	3	0	0	3	5	2	0	5	4	1	0	8	1	1	0	7.89 ± 0.34 ^c^
30	8	2	0	0	3	6	1	0	8	2	0	0	8	2	0	0	8.26 ± 0.24 ^ab^
40	9	1	0	0	5	4	1	0	8	2	0	0	9	1	0	0	8.46 ± 0.20 ^a^
50	8	2	0	0	5	5	0	0	8	2	0	0	7	3	0	0	8.38 ± 0.13 ^a^

E—excellent; G—good; M—moderate; P—poor. Different alphabet represents significant difference in the column (*p* < 0.05).

## Data Availability

Data is contained within the article and Supplementary Material.

## Supplementary Materials

The following supporting information can be downloaded at: https://www.mdpi.com/article/10.3390/foods11101504/s1, Table S1: Sensory scoring standard.

## References

[1] Kurt A., Atalar I. (2018). Effects of quince seed on the rheological, structural and sensory characteristics of ice cream. Food Hydrocoll..

[2] Akbari M., Eskandari M.H., Davoudi Z. (2019). Application and functions of fat replacers in low-fat ice cream: A review. Trends Food Sci. Technol..

[3] Singh H. (2019). Symposium review: Fat globules in milk and their structural modifications during gastrointestinal digestion. J. Dairy Sci..

[4] Zhang W., Zhao P., Li J., Wang X., Hou J., Jiang Z. (2022). Effects of ultrasound synergized with microwave on structure and functional properties of transglutaminase-crosslinked whey protein isolate. Ultrason. Sonochem..

[5] Huppertz T., Kelly A., Fox P., McSweeney P. (2006). Physical chemistry of milk fat globules. Advanced Dairy Chemistry.

[6] Wang W., Liu F., Xu C., Liu Z., Ma J., Gu L., Jiang Z., Hou J. (2021). *Lactobacillus plantarum* 69-2 combined with galacto-oligosaccharides alleviates d-galactose-induced aging by regulating the AMPK/SIRT1 signaling pathway and gut microbiota in mice. J. Agric. Food Chem..

[7] Liu Z., Zhao J., Sun R., Wang M., Wang K., Li Y., Shang H., Hou J., Jiang Z. (2022). Lactobacillus plantarum 23-1 improves intestinal inflammation and barrier function through the TLR4/NF-κB signaling pathway in obese mice. Food Funct..

[8] Xu C., Fu Y., Liu F., Liu Z., Ma J., Jiang R., Song C., Jiang Z., Hou J. (2021). Purification and antimicrobial mechanism of a novel bacteriocin produced by Lactobacillus rhamnosus 1.0320. LWT.

[9] Zhou X., Zhao J., Zhao X., Sun R., Sun C., Hou D., Zhang X., Jiang L., Hou J., Jiang Z. (2022). Oil bodies extracted from high-oil soybeans (Glycine max) exhibited higher oxidative and physical stability than oil bodies from high-protein soybeans. Food Funct..

[10] Zaaboul F., Zhao Q., Xu Y., Liu Y. (2022). Soybean oil bodies: A review on composition, properties, food applications, and future research aspects. Food Hydrocoll..

[11] Wang W., Cui C., Wang Q., Sun C., Jiang L., Hou J. (2019). Effect of pH on physicochemical properties of oil bodies from different oil crops. J. Food Sci. Technol..

[12] Chen Y., Cao Y., Zhao L., Kong X., Hua Y. (2014). Macronutrients and micronutrients of soybean oil bodies extracted at different pH. J. Food Sci..

[13] Nikiforidis C.V., Matsakidou A., Kiosseoglou V. (2014). Composition, properties and potential food applications of natural emulsions and cream materials based on oil bodies. RSC Adv..

[14] Jiang Z., Mu S., Ma C., Liu Y., Ma Y., Zhang M., Li H., Liu X., Hou J., Tian B. (2022). Consequences of ball milling combined with high-pressure homogenization on structure, physicochemical and rheological properties of citrus fiber. Food Hydrocoll..

[15] Zhao X., Wang K., Zhao J., Sun R., Shang H., Sun C., Liu L., Hou J., Jiang Z. (2022). Physical and oxidative stability of astaxanthin microcapsules prepared with liposomes. J. Sci. Food Agric..

[16] Pon S.Y., Lee W.J., Chong G.h. (2015). Textural and rheological properties of stevia ice cream. Int. Food Res. J..

[17] Horwitz W., Latimer, AOAC International (2019). Official method 2000.18. Official Methods of Analysis of AOAC International.

[18] Silva J.M., Klososki S.J., Silva R., Raices R.S.L., Silva M.C., Freitas M.Q., Barão C.E., Pimentel T.C. (2020). Passion fruit-flavored ice cream processed with water-soluble extract of rice by-product: What is the impact of the addition of different prebiotic components?. LWT.

[19] Zhou X., Liu Z., Wang W., Miao Y., Gu L., Li Y., Liu X., Jiang L., Hou J., Jiang Z. (2021). NaCl induces flocculation and lipid oxidation of soybean oil body emulsions recovered by neutral aqueous extraction. J. Sci. Food Agric..

[20] Ma Y., Liu Y., Yu H., Mu S., Li H., Liu X., Zhang M., Jiang Z., Hou J. (2022). Biological activities and in vitro digestion characteristics of glycosylated α-lactalbumin prepared by microwave heating: Impacts of ultrasonication. LWT.

[21] Hageman J.H.J., Keijer J., Dalsgaard T.K., Zeper L.W., Carrière F., Feitsma A.L., Nieuwenhuizen A.G. (2019). Free fatty acid release from vegetable and bovine milk fat-based infant formulas and human milk during two-phase in vitro digestion. Food Funct..

[22] Li J., Liu Y., Li T., Gantumur M.-A., Qayum A., Bilawal A., Jiang Z., Wang L. (2022). Non-covalent interaction and digestive characteristics between α-lactalbumin and safflower yellow: Impacts of microwave heating temperature. LWT.

[23] Nikiforidis C.V., Kiosseoglou V. (2009). Aqueous extraction of oil bodies from maize germ (Zea mays) and characterization of the resulting natural oil-in-water emulsion. J. Agric. Food Chem..

[24] Dogan M., Kayacier A., Toker Ö.S., Yilmaz M.T., Karaman S. (2013). Steady, dynamic, creep, and recovery analysis of ice cream mixes added with different concentrations of xanthan gum. Food Bioproc. Tech..

[25] Mostafavi F.S., Tehrani M.M., Mohebbi M. (2017). Rheological and sensory properties of fat reduced vanilla ice creams containing milk protein concentrate (MPC). J. Food Meas. Charact..

[26] Jirapeangtong K., Siriwatanayothin S., Chiewchan N. (2008). Effects of coconut sugar and stabilizing agents on stability and apparent viscosity of high-fat coconut milk. J. Food Eng..

[27] Bahramparvar M., Tehrani M.M. (2011). Application and functions of stabilizers in ice cream. Food Rev. Int..

[28] Soukoulis C., Tzia C. (2018). Grape, raisin and sugarcane molasses as potential partial sucrose substitutes in chocolate ice cream: A feasibility study. Int. Dairy J..

[29] Sun C., Wu T., Liu R., Liang B., Tian Z., Zhang E., Zhang M. (2015). Effects of superfine grinding and microparticulation on the surface hydrophobicity of whey protein concentrate and its relation to emulsions stability. Food Hydrocoll..

[30] Qian C., McClements D.J. (2011). Formation of nanoemulsions stabilized by model food-grade emulsifiers using high-pressure homogenization: Factors affecting particle size. Food Hydrocoll..

[31] Aboulfazli F., Baba A.S., Misran M. (2015). Effects of fermentation by *Bifidobacterium* bifidum on the rheology and physical properties of ice cream mixes made with cow and vegetable milks. Int. J. Food Sci. Technol..

[32] Liu R., Wang L., Liu Y., Wu T., Zhang M. (2018). Fabricating soy protein hydrolysate/xanthan gum as fat replacer in ice cream by combined enzymatic and heat-shearing treatment. Food Hydrocoll..

[33] Muzammil H.S., Rasco B., Sablani S. (2017). Effect of inulin and glycerol supplementation on physicochemical properties of probiotic frozen yogurt. Food Nutr. Res..

[34] Seo C.W., Oh N.S. (2022). Functional application of Maillard conjugate derived from a κ-carrageenan/milk protein isolate mixture as a stabilizer in ice cream. LWT.

[35] Lomolino G., Zannoni S., Zabara A., Da Lio M., De Iseppi A. (2020). Ice recrystallisation and melting in ice cream with different proteins levels and subjected to thermal fluctuation. Int. Dairy J..

[36] Chen W., Liang G., Li X., He Z., Zeng M., Gao D., Qin F., Goff H.D., Chen J. (2019). Effects of soy proteins and hydrolysates on fat globule coalescence and meltdown properties of ice cream. Food Hydrocoll..

[37] Javidi F., Razavi S.M.A., Behrouzian F., Alghooneh A. (2016). The influence of basil seed gum, guar gum and their blend on the rheological, physical and sensory properties of low fat ice cream. Food Hydrocoll..

[38] Wee M.S.M., Goh A.T., Stieger M., Forde C.G. (2018). Correlation of instrumental texture properties from textural profile analysis (TPA) with eating behaviours and macronutrient composition for a wide range of solid foods. Food Funct..

[39] Akbari M., Eskandari M.H., Niakosari M., Bedeltavana A. (2016). The effect of inulin on the physicochemical properties and sensory attributes of low-fat ice cream. Int. Dairy J..

[40] Kapchie V.N., Yao L., Hauck C.C., Wang T., Murphy P.A. (2013). Oxidative stability of soybean oil in oleosomes as affected by pH and iron. Food Chem..

[41] Speziali G., Liesinger L., Gindlhuber J., Leopold C., Pucher B., Brandi J., Castagna A., Tomin T., Krenn P., Thallinger G.G. (2018). Myristic acid induces proteomic and secretomic changes associated with steatosis, cytoskeleton remodeling, endoplasmic reticulum stress, protein turnover and exosome release in HepG2 cells. J. Proteom..

[42] Hernáez Á., Castañer O., Elosua R., Pintó X., Estruch R., Salas-Salvadó J., Corella D., Arós F., Serra-Majem L., Fiol M. (2017). Mediterranean diet improves high-density lipoprotein function in high-cardiovascular-risk individuals: A randomized controlled trial. Circulation.

[43] Kumar N.G., Contaifer D., Madurantakam P., Carbone S., Price E.T., Van Tassell B., Brophy D.F., Wijesinghe D.S. (2019). Dietary bioactive fatty acids as modulators of immune function: Implications on human health. Nutrients.

[44] Kenari R.E., Razavi R. (2021). Effect of sonication conditions: Time, temperature and amplitude on physicochemical, textural and sensory properties of yoghurt. Int. J. Dairy Technol..

[45] Çörekçi B., Toy E., ÖZTÜRK F., Malkoc S., Öztürk B. (2015). Effects of contemporary orthodontic composites on tooth color following short-term fixed orthodontic treatment: A controlled clinical study. Turk. J. Med. Sci..

[46] Yang C.J., Ding W., Ma L.J., Jia R. (2015). Discrimination and characterization of different intensities of goaty flavor in goat milk by means of an electronic nose. J. Dairy Sci..

[47] Silletti E., Vingerhoeds M.H., Norde W., van Aken G.A. (2007). The role of electrostatics in saliva-induced emulsion flocculation. Food Hydrocoll..

[48] Sarkar A., Goh K.K.T., Singh R.P., Singh H. (2009). Behaviour of an oil-in-water emulsion stabilized by β-lactoglobulin in an in vitro gastric model. Food Hydrocoll..

[49] Clulow A.J., Salim M., Hawley A., Boyd B.J. (2018). A closer look at the behaviour of milk lipids during digestion. Chem. Phys. Lipids.

[50] Li M., Liu Y., Zhao J., Yu R., Hussain M.A., Qayum A., Jiang Z., Qu B. (2022). Glycosylated whey protein isolate enhances digestion behaviors and stabilities of conjugated linoleic acid oil in water emulsions. Food Chem..

[51] Dai C., Zhang W., He R., Xiong F., Ma H. (2017). Protein breakdown and release of antioxidant peptides during simulated gastrointestinal digestion and the absorption by everted intestinal sac of rapeseed proteins. LWT.

