# Supplementary material for: Effects of Soybean Oil Body as a Milk Fat Substitute on Ice Cream: Physicochemical, Sensory and Digestive Properties
Source: Foods. 2022 May 22;11(10):1504. doi: 10.3390/foods11101504 (PMC9141774; doi:10.3390/foods11101504)
Supplement: Supplementary file 1 [file foods-11-01504-s001.zip › foods-1725519-supplementary.pdf]

Article

Effects of soybean oil body as a milk fat substitute on ice cream: physicochemical, sensory and digestive properties

Wan Wang [1], Min Wang [1], Cong Xu [1], Zhijing Liu [1], Liya Gu [1], Jiage Ma [1], Lianzhou Jiang [1], Zhanmei Jiang [1] and Juncai Hou [1,*]

[1] College of Food Science, Northeast Agricultural University, Harbin 150030, China; 13159806631@163.com (W.W.); 18246037810@163.com (M.W.); 15636116265@163.com (C.X.); liuzhijing22@126.com (Z.L.); Andrea_hh@163.com (L.G.); jiage_ma@neau.edu.cn (J.M.); jlzname@neau.edu.cn (J.L.); zhanmeijiang@neau.edu.cn (Z.J.)

* Correspondence: jchou@neau.edu.cn (J.H.); Tel.: +86-451-55190710

Table S1. Sensory scoring standard.

Grade	Color	Taste	Texture	Flavor
Excellent (8-10)	Uniform color, milky white, without variegation	The delicate and lubricating taste, the moderate sweetness	Complete, not collapsed, no obvious ice crystals	Rich milk flavor and bean flavor, no odor
Good (6-8)	Relatively uniform color, milky white, without variegation	The finely lubricated taste, slightly sweet or light sweetness	Relatively complete, slightly collapsed, no obvious ice crystals	Milk flavor and bean flavor, a little odor
Moderate (4-6)	Uneven color, milky white, slightly mixed color	Not delicate and lubricated taste, too sweet or light sweetness	Relatively complete, slightly collapsed, a little ice crystals	A little milk flavor and bean flavor, slight odor
Poor (2-4)	Uneven color, deep or shallow milky white, obvious variegated	Not delicate and not lubricated taste, too sweet or light sweetness	Not complete, collapsed, lots of ice crystals	No milk flavor and bean flavor, obvious odor
